# RETRACTION: Wound Dehiscence in Enhanced Recovery After OpenRadical Cystectomy: A Meta‐Analysis

**DOI:** 10.1111/iwj.70232

**Published:** 2025-02-11

## Abstract

**RETRACTION:** XueG. , LiuY. , JiX. , and ZhangH. , “Wound Dehiscence in Enhanced Recovery After Open Radical Cystectomy: A Meta‐Analysis,” International Wound Journal 20, no. 7 (2023): 2634–2639, 10.1111/iwj.14136.36880410 PMC10410360

The above article, published online on 07 March 2023, in Wiley Online Library (http://onlinelibrary.wiley.com/), has been retracted by agreement between the journal Editor in Chief, Professor Keith Harding; and John Wiley & Sons Ltd. Following an investigation by the publisher, all parties have concluded that this article was accepted solely on the basis of a compromised peer review process. The editors have therefore decided to retract the article. The authors did not respond to our notice regarding the retraction.

**RETRACTION:** G. Xue , Y. Liu , X. Ji , and H. Zhang , “Wound Dehiscence in Enhanced Recovery After Open Radical Cystectomy: A Meta‐Analysis,” International Wound Journal 20, no. 7 (2023): 2634–2639, 10.1111/iwj.14136.36880410 PMC10410360

The above article, published online on 07 March 2023, in Wiley Online Library (http://onlinelibrary.wiley.com/), has been retracted by agreement between the journal Editor in Chief, Professor Keith Harding; and John Wiley & Sons Ltd. Following an investigation by the publisher, all parties have concluded that this article was accepted solely on the basis of a compromised peer review process. The editors have therefore decided to retract the article. The authors did not respond to our notice regarding the retraction.

